# Rationally seeded computational protein design of ɑ-helical barrels

**DOI:** 10.1038/s41589-024-01642-0

**Published:** 2024-06-20

**Authors:** Katherine I. Albanese, Rokas Petrenas, Fabio Pirro, Elise A. Naudin, Ufuk Borucu, William M. Dawson, D. Arne Scott, Graham. J. Leggett, Orion D. Weiner, Thomas A. A. Oliver, Derek N. Woolfson

**Affiliations:** 1 School of Chemistry, University of Bristol, Bristol, UK; 2 Max Planck-Bristol Centre for Minimal Biology, University of Bristol, Bristol, UK; 3 School of Biochemistry, University of Bristol, Medical Sciences Building, Bristol, UK; 4 Rosa Biotech, Science Creates St Philips, Bristol, UK; 5 Department of Chemistry, University of Sheffield, Sheffield, UK; 6 Cardiovascular Research Institute, Department of Biochemistry and Biophysics, University of California San Francisco, San Francisco, CA USA; 7 Bristol BioDesign Institute, University of Bristol, Bristol, UK

**Keywords:** Protein design, Protein design

## Abstract

Computational protein design is advancing rapidly. Here we describe efficient routes starting from validated parallel and antiparallel peptide assemblies to design two families of α-helical barrel proteins with central channels that bind small molecules. Computational designs are seeded by the sequences and structures of defined de novo oligomeric barrel-forming peptides, and adjacent helices are connected by loop building. For targets with antiparallel helices, short loops are sufficient. However, targets with parallel helices require longer connectors; namely, an outer layer of helix–turn–helix–turn–helix motifs that are packed onto the barrels. Throughout these computational pipelines, residues that define open states of the barrels are maintained. This minimizes sequence sampling, accelerating the design process. For each of six targets, just two to six synthetic genes are made for expression in *Escherichia coli*. On average, 70% of these genes express to give soluble monomeric proteins that are fully characterized, including high-resolution structures for most targets that match the design models with high accuracy.

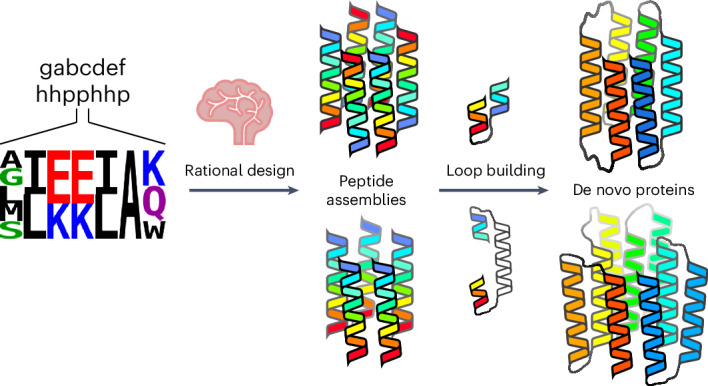

## Abstract (web summary)

An efficient computational pipeline starting from validated peptide assemblies has been used to design two families of α-helical barrel proteins with functionalizable channels. This rationally seeded computational protein design approach delivers soluble, monomeric proteins that match the design targets accurately and with high success rates.

## Main

Approaches to de novo protein design have developed considerably over the past four decades^1–5^. Early in the field of protein design, minimal design used straightforward chemical principles, particularly the patterning of hydrophobic and polar residues, to deliver peptide assemblies and relatively simple protein architectures. Largely, this gave way to rational design, in which sequence design was augmented by understood sequence-to-structure relationships garnered from bioinformatics and biochemical experiments. This delivered more varied and more robust designs. In parallel, computational design emerged, allowing the realization of concepts such as fragment-based and parametric backbone design, and methods for fitting de novo sequences onto these scaffolds^2,6,7^. In turn, this has led to increasingly complex designs of new structures and functions for both water-soluble and membrane-spanning proteins^3^. Currently, the field is undergoing another step change with the application of data-driven and deep learning methods to generate de novo protein sequences, structures and functions^5,8–18^. These methods have the potential to democratize protein design^11,19^ and to promote its application in biotechnology^20,21^, cell biology^22^, materials science^23,24^ and medicine^25–27^.

Despite this progress, considerable challenges remain to realize the full promise of de novo protein design, both in terms of advancing fundamental protein science and making it a robust and reliable alternative to engineering natural proteins for the application areas listed above. Current challenges include generating starting backbones that can be designed^11,28,29^ to achieve a desired function, and increasing the success rates of converting in silico designs into experimentally confirmed proteins^8,30–32^. In addition to these practical issues, we must address the concern that although deep learning approaches will continue to advance our abilities to design protein structures and functions in new and unforeseen ways, it is less clear that they will necessarily improve our basic understanding of protein structure and function. Here, to bridge this gap, we advocate for and demonstrate the potential of combining rational and computational protein design. Specifically, we use understood sequence-to-structure relationships for α-helical peptide assemblies to seed the computational design of single-chain proteins, which are completed by loop building using advanced computational methods, including deep learning approaches. In this way, we deliver robust new protein sequences and structures—namely, barrel-like proteins with accessible and functionalizable central channels—rapidly and with high success rates.

**Fig. 1:**
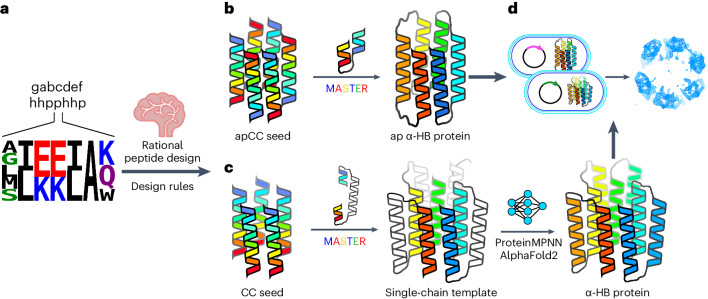
Pipeline for rationally seeded computational design of de novo protein folds. **a**, Robust sequence-to-structure relationships for coiled-coil oligomers were used as rules to seed the design of new protein scaffolds. **b**,**c**, Antiparallel (**b**) and parallel (**c**) α-helical barrel protein design targets. For both targets, MASTER^51,52^ was used to search known experimental protein structures for segments with the potential to connect adjacent helices and generate single-chain models. For the antiparallel designs (**b**), the sequences and structures of identified short connectors were used directly. However, the parallel targets required longer structured loops (**c**), for which we targeted helix–turn–helix–turn–helix motifs. ProteinMPNN^8^ and AlphaFold2 (refs. ^55,56^) were then used iteratively to optimize the sequences and models of these three-helix bundle motifs. **d**, For each design, a small number of synthetic genes were made and expressed in *E. coli* for biophysical and structural characterization. Peptide and protein chains are shown in chainbows from the N termini to the C termini (blue to red), except for the initially placed central helices of the helix–turn–helix–turn–helix motifs in the parallel designs, which are shown in white. α-HB, α-helical barrel.

Over the past decade, a range of oligomeric α-helical barrels have been designed based on self-assembling peptides that encode highly specific and stable coiled-coil interactions^33,34^. These α-helical barrel peptides are interesting de novo scaffolds because of their stability, robustness to mutation and potential to functionalize their internal lumens^20,35–37^. However, the scope for developing these is limited because they are peptide-based and largely homo-oligomeric. Thus, any changes made to the peptide sequences are repeated symmetrically in each peptide of the assembly. One solution to increase the utility of α-helical barrels is to connect the helices to form single polypeptide chains that can be produced by the expression of synthetic genes. Symmetry can then be broken with mutations in individual helices of the structure. However, connecting the helices is not straightforward, as the majority of α-helical barrels presented so far have all-parallel helices. Here we describe two routes to design α-helical barrel proteins. In the first, we design new antiparallel α-helical barrel peptide assemblies and then connect adjacent helices to form single chains using short loops (Fig. 1b). Second, for existing all-parallel α-helical barrel peptides, the helices are connected by longer structured loops (Fig. 1c). In both cases, we test several approaches to computational loop building. A key aspect of our design process is that it uses validated sequence-to-structure relationships garnered from the oligomeric peptides as rules to seed the designs rather than designing entirely new sequences. This speeds up the design process, produces robust in silico models, limits the number of constructs tested and yields high success rates of experimentally confirmed targets (Fig. 1d).

## Results

### New peptide rules deliver rarer antiparallel α-helical barrels

**Fig. 2:**
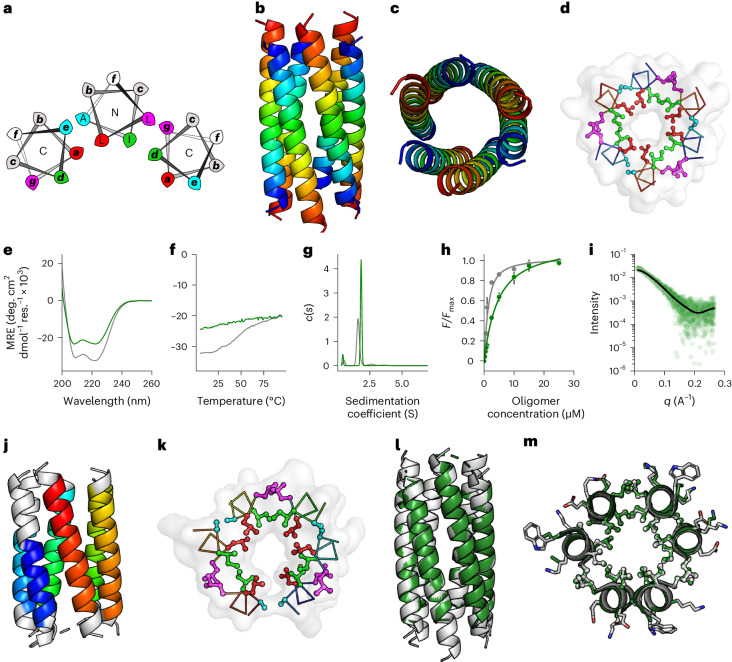
Biophysical and structural characterization of the apCC-Hex peptide and the sc-apCC-6-LLIA protein. **a**, Helical-wheel representation of part of an antiparallel α-helical barrel highlighting the ***a***–***g*** heptad repeats: red, ***a*** sites; green, ***d*** sites; magenta, ***g*** sites; and cyan, ***e*** sites; N and C labels refer to the termini of the helices closest to the viewer. **b**–**d**, X-ray crystal structure (1.4-Å resolution) of apCC-Hex (PDB ID, 8QAB). Coiled-coil regions identified by Socket2 (ref. ^72^) (packing cutoff, 7.0 Å) are colored as chainbows from N termini to C termini (blue to red) (**b**,**c**). **d**, A slice through the structure of a heptad repeat with KIH packing colored the same as in the helical wheel in **a**. **e**–**h**, Comparison of the biophysical data for the apCC-Hex α-helical barrel peptide (gray) and the sc-apCC-6-LLIA α-helical barrel protein (green). Circular dichroism spectra were recorded at 5 °C (**e**). **f**, Thermal responses of the α-helical circular dichroism signal at 222 nm. **g**, AUC sedimentation velocity data at 20 °C are fitted to a single-species model; fits returned a peptide assembly of 18.7 kDa (hexamer) and a protein of 24.0 kDa (monomer). **h**, Fitted data for DPH binding to the peptide and protein; fits returned dissociation constant (*K*_d_) values of 0.8 ± 0.3 µM and 4.0 ± 0.4 µM, respectively. Fitted data are the mean and s.d. of three independent repeats. **i**, SEC-SAXS data for sc-apCC-6-LLIA fitted using FoXS^57,58^ to an AlphaFold2 model of the design (*χ*^2^ = 1.50). **j**, X-ray crystal structure (2.25 Å) of sc-apCC-6-LLIA (PDB ID, 8QAD) with coiled-coil regions identified by Socket2 (ref. ^72^) (packing cutoff, 7.0 Å) colored as chainbows. **k**, A slice through the structure of a heptad repeat showing KIH packing, colored as in **a**. **l**,**m**, Overlays of the experimental apCC-Hex (gray) and sc-apCC-6-LLIA protein (green) structures (RMSD for backbone atoms (RMSD_bb_) = 1.177 Å). The conditions were as follows: circular dichroism spectroscopy, 50 µM peptide, 10 µM protein in PBS, pH 7.4; AUC, 100 µM peptide, 15 µM protein in PBS, pH 7.4; DPH binding, oligomer concentration was 0–30 µM peptide, 0–30 µM protein in PBS, pH 7.4, 20 °C, final concentration was 1 µM DPH (5% v/v DMSO); SEC-SAXS, 10 mg ml^−1^ protein in PBS, pH 7.4. deg., degrees; MRE, mean residue ellipticity; res., residue. Source data

So far, most α-helical barrel peptides have all-parallel arrangements of helices^34^. Given the extended connections required (Fig. 1c), turning these into single-chain α-helical barrel proteins is not trivial. Conversely, α-helical barrel peptides with adjacent antiparallel helices could be converted to α-helical barrel proteins using short linkers between helices (Fig. 1b). However, antiparallel α-helical barrel peptides are less common^38–40^ and therefore present their own design challenge. Hence, to initiate our peptides-to-proteins approach, we tested an informed subset of synthetic peptides based on the collective understanding of coiled coils^34^ that could potentially form homomeric antiparallel hexameric α-helical barrels. Our designs focused on the ***g****-****a****-****d****-****e*** sites of the classical coiled-coil heptad sequence repeat ***gabcdef***, as these sites contribute most to the helix–helix interfaces (Fig. 2a). Specifically, we investigated 20 sequence combinations in which ***g*** = Ala, Gly, Leu, Met or Ser, and ***a*** and ***d*** = Ile or Leu. AlphaFold2-multimer predictions of six-peptide oligomers suggested that 19 out of 20 of these sequences should form open, α-helical barrels (Supplementary Figs. 1 and 2). With these models and our understanding of coiled coils in mind, the sequence combinations were installed into four-heptad peptide sequences with a common background comprising ***e*** = Ala^40–42^, a ‘bar-magnet’ charge patterning of Glu and Lys at ***b*** and ***c*** to favor antiparallel coiled-coil assemblies^40,42,43^, and ***f*** = Gln, Lys and Trp to aid helicity and solubility, and to add a chromophore. The 20 sequences (Supplementary Table 1) were made by solid-phase peptide synthesis, purified by high-performance liquid chromatography (HPLC) and confirmed by mass spectrometry (Supplementary Fig. 3). Each peptide was tested for α-helicity and thermal stability by circular dichroism spectroscopy (Fig. 2e,f and Supplementary Figs. 4 and 5) and for oligomeric state by analytical ultracentrifugation (AUC) (Fig. 2g, Supplementary Table 2 and Supplementary Figs. 6 and 7). Fourteen of these sequences formed hyperstable, helical hexamers (Supplementary Table 3).

To test which of these peptides formed barrel-like and potentially functionalizable structures, we used the environment-sensitive dye 1,6-diphenyl hexatriene (DPH), which fluoresces when in hydrophobic environments like the lumens of open α-helical barrels. We have shown that low micromolar DPH binding provides a solution-phase proxy for open-barrel states observed by X-ray crystallography^36^, and that it can be used as a reporter in α-helical barrel sensing assays^20^. On this basis, 14 of the peptides tested were assessed as potentially having accessible central channels (Supplementary Table 3 and Supplementary Fig. 8).

We solved high-resolution X-ray crystal structures of three peptides using ab initio phasing^44,45^. One structure, with ***g***-***a***-***d***-***e*** = Ala-Leu-Ile-Ala, revealed an antiparallel hexamer consistent with its solution-phase oligomer state (Supplementary Table 2). However, this was a collapsed bundle, conflicting with the solution-phase binding data that suggest that this peptide can access an open α-helical barrel (Supplementary Table 3 and Supplementary Fig. 10). Another structure, with ***g***-***a***-***d***-***e*** = Gly-Leu-Ile-Ala, had promising solution-phase data for an open hexamer or heptamer (Supplementary Tables 2 and 3), but, interestingly, formed a collapsed antiparallel octamer in the crystal state (Supplementary Fig. 11). Some plasticity in assemblies formed from these types of peptides is expected^46^. Also, we have reported a parallel α-helical barrel that accesses both an open barrel and a collapsed bundle in the crystal state but still binds DPH with low micromolar affinities^47^. Thus, it is possible that Ala-Leu-Ile-Ala and Gly-Leu-Ile-Ala can also access an open conformation in solution. Indeed, DPH binding by these peptide assemblies is patently different from the control, CC-Tri (a homomeric3-helix bundle in solution and in the crystal state), which does not bind DPH^36^ (Supplementary Fig. 8). However, and by contrast, the X-ray crystal structure of ***g***-***a***-***d***-***e*** = Leu-Leu-Ile-Ala revealed the targeted antiparallel hexameric open barrel with completely consistent solution-phase behavior^40^ (Fig. 2b–d, Supplementary Table 3 and Supplementary Fig. 12). We named this peptide apCC-Hex-LLIA, and systematically as apCC-Hex.

In summary, after filtering at each stage of solution-phase biophysical and structural characterization, of the 20 initial starting sequences, 12 (60%) were promising for taking forward to design single-chain proteins (Supplementary Fig. 9). This process illustrates the importance of establishing robust rules for the next stage of the protein design pipeline.

### Short loops yield an antiparallel α-helical barrel protein

Using the experimental apCC-Hex structure as a seed, we designed short loop sequences computationally to connect adjacent helices to generate an up-down α-meander structure (Fig. 1b). We tested three approaches. First, and most simply, we took loops from the literature to span the distances between the carboxyl and amino termini of the helices^40,48–50^. Secondly, we used the ColabPaint implementation of Protein Inpainting^9^ to hallucinate loop sequences (https://github.com/polizzilab/design_tools). Finally, we applied MASTER^51,52^ to find tertiary fragments that link the helices (Supplementary Table 4). Given two fragments, MASTER performs backbone alignments to find target structures from the Research Collaboratory for Structural Bioinformatics Protein Data Bank (RCSB PDB) that best match the query fragments. This approach has been used successfully to connect α-helices and β-strands^53,54^. The resulting single-chain templates were used in a computational screen to find the best-fitting combinations of residues at the ***g***-***a***-***d*** sites (with ***e*** sites fixed as Ala). This was guided by the privileged residue combinations from the experiments with synthetic peptides (Supplementary Table 3). Models for these ***g***-***a***-***d*** combinations with different loop sequences were built using AlphaFold2 (refs. ^55,56^) in single-sequence mode (Supplementary Figs. 9 and 13–15) and assessed by predicted local distance difference test (pLDDT) from AlphaFold2 and root mean squared deviation (RMSD) to the parent apCC-Hex starting scaffold. In this way, we generated seven sequences with different ***g***-***a***-***d***-***e*** combinations and loop-building methods (Supplementary Tables 5 and 6 and Supplementary Fig. 9).

Synthetic genes for all except two of the seven sequences expressed in *E. coli* (Supplementary Tables 6–8). As the peptide assemblies were hyperthermally stable, we heat treated the cell lysate (75 °C for 10 min) and subjected the soluble fraction to immobilized metal affinity chromatography (IMAC) and size exclusion chromatography (SEC) to yield highly pure proteins in a minimal number of steps (Supplementary Fig. 16). Circular dichroism spectroscopy showed that all five proteins were α-helical and hyperthermally stable structures (Fig. 2e,f and Supplementary Figs. 17 and 18), and AUC confirmed that they were monomers (Fig. 2g, Supplementary Table 7 and Supplementary Fig. 19). Moreover, DPH binding suggested that they had accessible hydrophobic channels (Fig. 2h and Supplementary Fig. 19). These data (Supplementary Table 8) were supported by SEC coupled with small-angle X-ray scattering (SEC-SAXS) data, which fitted to their respective AlphaFold2 models with good *χ*^2^ values^57,58^ (Fig. 2i, Supplementary Table 9 and Supplementary Fig. 21). Finally, we obtained two high-resolution X-ray crystal structures using ab initio phasing and molecular replacement for sequences generated using MASTER^51,52^: one was directly derived from apCC-Hex, ***g***-***a***-***d***-***e*** = Leu-Leu-Ile-Ala (Fig. 2j–m and Supplementary Fig. 22), and the other, ***g***-***a***-***d***-***e*** = Ser-Leu-Leu-Ala, was one of the tighter dye-binding proteins that was characterized (Supplementary Fig. 23). The sequences and structures were named sc-apCC-6-LLIA and sc-apCC-6-SLLA, respectively, for single-chain antiparallel coiled-coil proteins with six central helices.

Thus, the success rate for making these single-chain constructs from the seven antiparallel designs test was five soluble proteins (71%) and two new α-helical barrel crystal structures (29%) (Supplementary Fig. 9).

### Structured α-helical motifs link parallel helices

The parallel α-helical barrel proteins required a different design approach, as sequence-to-structure relationships for the ***g***-***a***-***d***-***e*** positions were available to seed the designs^33,46,59^, but connecting adjacent parallel helices was not straightforward because of the need to span ~40 Å along the structures (Fig. 1c). Indeed, previously we had made several unsuccessful attempts to link parallel helices using polyproline helix-based linkers^60^. Therefore, we tested whether MASTER^51,52^ could find better α-helical templates from the PDB to address this. We exploited the *C*_n_ symmetry of the parallel α-helical barrel peptides to generate helix–turn–helix–turn–helix units, which could be repeated about the *C*_n_ axis to close structures with *n* central helices and *n*−1 buttressing helices (Fig. 1c). To find helix–turn–helix–turn–helix units, we queried the adjacent helices from crystal structures of parallel α-helical barrels against a nonredundant set of three-helix coiled-coil bundles from the CC+ database^61,62^. This delivered several candidate backbones from which we chose the lowest RMSD hit for each target (Supplementary Table 4). A key advantage of MASTER is that the target backbone comes from an experimental structure and, hence, is inherently designable. This compares favorably to more computationally intensive tools that require large sampling to optimize backbone geometries^10,11^.

**Fig. 3:**
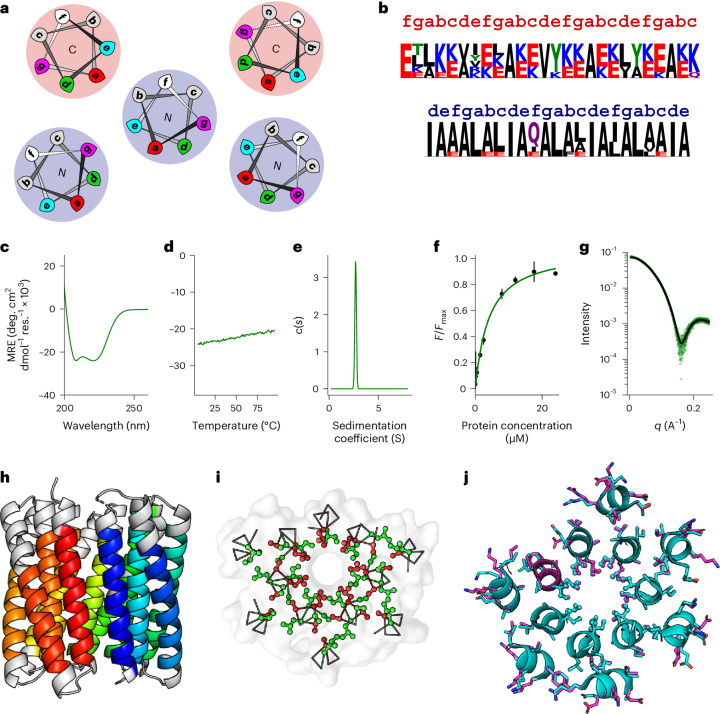
Biophysical and structural characterisation of sc-CC-7 de novo proteins. **a**, Helical-wheel representation for part of a parallel single-chain α-helical barrel showing KIH packing for the buttressing helices (shaded red) and the inner barrel (shaded blue): red, ***a*** sites; green, ***d*** sites; magenta, ***g*** sites; and cyan, ***e*** sites; N and C labels refer to the termini of the helices closest to the viewer. **b**, Sequence pileups and registers for the inner (blue register) and buttressing (red register) helices of sc-CC-7-LI. **c**,**d**, Circular dichroism spectrum recorded at 5 °C (**c**) and thermal-response curve (**d**) for sc-CC-7-LI. **e**, AUC sedimentation velocity data for sc-CC-7-LI fitted to a single-species model, which returned *M*_W_ = 37.4 kDa (monomer). **f**, Fitted binding data of DPH to sc-CC-7-LI, which returned *K*_d_ = 3.8 ± 0.8 µM. Fitted data are the mean and s.d. of three independent repeats. **g**, SEC-SAXS data fitted using the final AlphaFold2 model and FoXS (*χ*^2^ = 1.43)^57,58^. **h**, X-ray crystal structure of sc-CC-7-LI at a 2.5-Å resolution (PDB ID, 8QAI). Coiled-coil regions identified by Socket2 (ref. ^72^) (packing cutoff, 7 Å) are colored as chainbows from N termini to C termini (blue to red). **i**, A slice through the structure of a heptad repeat showing KIH packing with ***a***-type (red) and ***d****-*type (green) knobs. **j**, Overlay of the middle helical turns from the sc-CC-7-LI structure (cyan) and the final AlphaFold2 model (magenta) (RMSD_bb_ = 0.433 Å). The conditions were as follows: circular dichroism spectroscopy, 5 µM protein in PBS, pH 7.4; AUC, 25 µM protein in PBS, pH 7.4; DPH binding, 0–24 µM protein in PBS, pH 7.4, final concentration was 0.5 µM DPH (5% v/v DMSO); SEC-SAXS, 10 mg ml^−1^ protein in PBS, pH 7.4. Source data

Adding sequences to the new backbones required optimization of side-chain interactions in both the external three-helix bundle and the internal barrel (Fig. 3a). For the latter, again, sequence-to-structure relationships from existing α-helical barrel peptides seeded and accelerated sequence design. This is best illustrated by example (Supplementary Fig. 25). For instance, the ***g***-***a***-***d***-***e*** combination Ala-Leu-Ile-Ala defines the parallel heptamer CC-Hept (PDB ID, 4PNA)^33^. Therefore, these positions were fixed in the seven parallel inner helices of a 13-helix template derived from the backbone-generation procedure (Figs. 1c and 3b). Initially, the rest of the sequence was optimized using ProteinMPNN^8^. However, as others report^63^, we found that this placed hydrophobic residues on the solvent-exposed surface of the structure. To remedy this, as the outer helices were also based on coiled coils, we fixed the exposed ***b***, ***c*** and ***f*** sites to combinations of Glu, Lys and Gln (Supplementary Fig. 26). Initially, 100 sequences were generated, filtered based on core packing, Rosetta energy and charge, and modeled with AlphaFold2 (refs. ^55,56^) (Supplementary Fig. 25). The model with the best pLDDT score was used to initiate another round of sequence design. At this point, we replaced the fixed constraint on the outermost ***b***-***c***-***f*** residues with a Lys or Glu bias in ProteinMPNN^8^, followed by a surface hydrophobicity filter within Rosetta. This gave similar charge distributions and exposed hydrophobic scores but allowed less repetitive sequences to be generated (Supplementary Fig. 27). Iterations were repeated until the energies and the RMSDs between the ProteinMPNN^8^ inputs and the AlphaFold2 (refs. ^55,56^) outputs converged (Supplementary Fig. 27). For the sc-CC-7 target, this occurred after three rounds to yield helical sequences (Fig. 3b).

We chose four protein sequences with <85% sequence identity, high pLDDT and low Rosetta energies for gene synthesis and expression in *E. coli* (Supplementary Tables 10 and 11). Two of these sequences expressed. As for the antiparallel designs, these were purified by heat treatment, centrifugation, and IMAC and SEC to render highly pure protein (Supplementary Fig. 28). One of these (sc-CC-7-80) was oligomeric by AUC, which, although helical and thermally stable, was not characterized further (Supplementary Tables 12 and 13, and Supplementary Figs. 29–33). The other protein, named sc-CC-7-LI because of its ***a*** = Leu and ***d*** = Ile core, was helical and fully resistant to heat denaturation as judged by circular dichroism spectroscopy (Fig. 3c,d, Supplementary Table 13 and Supplementary Figs. 29 and 30), was monomeric according to AUC (Fig. 3e, Supplementary Table 12 and Supplementary Fig. 31) and bound dye, consistent with an accessible channel (Fig. 3f, Supplementary Table 13 and Supplementary Fig. 32). This was supported by SEC-SAXS data fit to the AlphaFold2 model^57,58^ (Fig. 3g, Supplementary Table 14 and Supplementary Fig. 33). We solved a 2.5-Å resolution X-ray structure by molecular replacement using the AlphaFold2 model for sc-CC-7-LI (Fig. 3h–j). Finally, to test the robustness of the design to mutation, we substituted all 49 ***a*** (Leu) and ***d*** (Ile) sites of the central α-helical barrel for alternative design rules for parallel heptameric α-helical barrels (that is, ***a*** = Ile and = Val)^46^. This protein (sc-CC-7-IV) was highly expressed and was also folded, as shown by circular dichroism spectroscopy and SEC-SAXS, hyperstable, monomeric and bound the reporter dye (Supplementary Tables 10–14 and Supplementary Figs. 28–33).

The success rate for making single-chain constructs from these initial five parallel designs was three soluble proteins (60%) and one new α-helical barrel crystal structure (20%).

### Seeded design rapidly accesses more α-helical barrel proteins

Encouraged by the successful design of sc-apCC-6 and sc-CC-7, we extended the seeded design approaches to target α-helical barrel proteins with five, six and eight central helices (Supplementary Tables 15–28 and Supplementary Figs. 34–68).

To seed the antiparallel eight-helix α-helical barrel protein design, we started with two sequences: the aforementioned peptide with ***g***-***a***-***d***-***e*** = Gly-Leu-Ile-Ala, which formed a collapsed antiparallel eight-helix bundle, and, from a previous study, ***g***-***a***-***d***-***e*** = Ala-Ile-Ile-Ala, with a different ***b*-*c*-*f*** background that forms an open parallel octamer by X-ray crystallography^59^. Therefore, we extended the peptide screen introduced above to explore this sequence space (Supplementary Table 1 and Supplementary Fig. 9). The resulting synthetic peptides formed stable, helical, higher-order oligomers with accessible channels (Supplementary Table 3 and Supplementary Figs. 3–9). Attempts to obtain diffraction-quality peptide crystals for these sequences were unsuccessful. Therefore, we used AlphaFold2 (refs. ^55,56^) to generate antiparallel octameric models to use as seeds for the computational design of single-chain antiparallel eight-helix α-helical barrel proteins (Supplementary Fig. 2). We used MASTER^51,52^ to find backbones to connect the helices (Supplementary Table 4). Next, ProteinMPNN^8^ was used to generate loop sequences, keeping the helical residues fixed and iterating with AlphaFold2 (refs. ^55,56^) to find sequences and models that were open α-helical barrels with the highest pLDDT. This led to two designs: ***g***-***a***-***d***-***e*** = Ala-Ile-Ile-Ala and ***g***-(***a***-***d***)_2_(***a***-***d***)_2_-***e*** = Gly-(Ile-Leu)_2_(Leu-Ile)_2_-Ala (Supplementary Tables 15 and 16, and Supplementary Figs. 9, 34 and 35). In the latter, two ***a***–***d*** combinations are repeated through the first two and last two heptads.

**Fig. 4:**
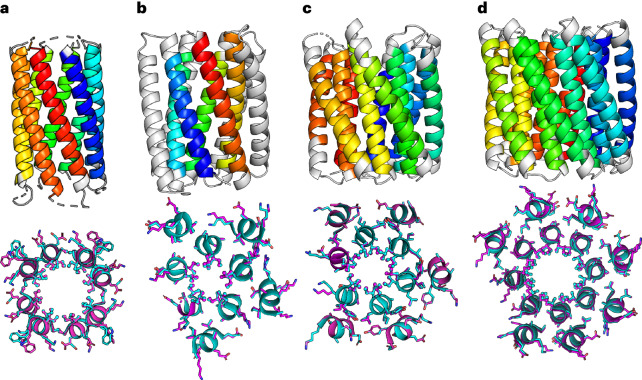
Structural characterization of five-helix, six-helix and eight-helix targets. **a**–**d**, Top, X-ray crystal structures of sc-apCC-8 at a 2.0-Å resolution (PDB ID, 8QAF) (**a**), sc-CC-5 at a 1.9-Å resolution (PDB ID, 8QKD) (**b**), sc-CC-6-95 at a 2.8-Å resolution (PDB ID, 8QAG) (**c**) and sc-CC-8-58 at a 2.35-Å resolution (PDB ID, 8QAH) (**d**). Coiled-coil regions identified by Socket2 (ref. ^72^) (packing cutoff, 7.5 Å for sc-apCC-8, sc-CC-5-24, sc-CC-6-95 and sc-CC-8-58 at 7.0 Å) are colored as chainbows from N termini (blue) to C termini (red). Bottom, overlays for the middle helical turns of each crystal structure (cyan) and the corresponding AlphaFold2 (refs. ^55,56^) model (magenta); RMSD_bb_ = 0.413 Å (**a**), RMSD_bb_ = 0.371 Å (**b**), RMSD_bb_ = 0.300 Å (**c**) and RMSD_bb_ = 0.530 Å (**d**). Source data

Both of these sequence designs expressed (Supplementary Fig. 36), and the purified proteins were soluble, folded, thermally stable, monomeric and monodisperse, with accessible cavities (Supplementary Tables 17 and 18, and Supplementary Figs. 37–40). This was confirmed by SEC-SAXS and X-ray crystallography (Fig. 4, Supplementary Table 19 and Supplementary Figs. 41 and 42). A 2.0-Å X-ray crystal structure was solved by ab initio phasing for ***g***-***a***-***d***-***e*** = Ala-Ile-Ile-Ala, which we called sc-apCC-8 (Fig. 4a and Supplementary Fig. 42).

For α-helical barrel proteins with inner barrels of five, six and eight parallel helices, we used seeds from existing peptide assemblies, with a modification of the six-helix target CC-Hex2 (PDB ID, 4PN9) to replace ***g*** = Ser in the peptide assembly with Ala to avoid polar Ser at the helix–turn–helix–turn–helix interface^33,46,59^ (Supplementary Tables 4, 20–28 and Supplementary Figs. 44–48). MASTER selected a similar right-handed helix–turn–helix–turn–helix tertiary fragment to connect the helices of the six- and eight-helix targets, as it did for sc-CC-7 (Supplementary Table 4), specifically, from a de novo helical repeat protein (PDB ID, 5CWQ)^64^. However, and interestingly, for the five-helix target, it returned a left-handed tertiary helix–turn–helix–turn–helix template from the same design series (PDB ID, 5CWI)^64^ (Supplementary Table 4). This can be rationalized because lower-order coiled-coil oligomers have clear left-handed, superhelical twists, whereas the larger helical assemblies have straighter superhelices^33,59,65^. For the three targets, 11 sequences were tested experimentally (Supplementary Tables 20–28 and Supplementary Figs. 49–66). Synthetic genes for all but two of these sequences expressed in *E. coli* and yielded soluble proteins that were α-helical, monomeric and thermally stable (Supplementary Figs. 49–66). The five-helix-based proteins showed no dye binding, although an X-ray crystal structure revealed an open barrel. Thus, the cavities of five-helix-based barrels appear to be too narrow to accommodate dye (Fig. 4, Supplementary Table 27 and Supplementary Fig. 53). By contrast, the six- and eight-helix-based targets bound dye, consistent with accessible cavities, which were confirmed by SEC-SAXS and X-ray crystal structures solved using molecular replacement (Fig. 4, Supplementary Tables 27 and 28, and Supplementary Figs. 55 and 66). Together, these additional designs delivered the de novo proteins sc-CC-5, sc-CC-6 and sc-CC-8.

In summary, from 13 designs, the success rate for making further single-chain proteins was 11 soluble proteins (78%) and four new α-helical barrel crystal structures (31%).

### The α-helical barrel proteins match the seeds and design models

We compared our experimental structures to the seed structures^33,59^, the utilized tertiary fragments^64^, and the final in silico design models generated by AlphaFold2 (refs. ^55,56^) (Supplementary Table 32). Because of changes from the full sequence-design steps, we compared backbone atoms only. Apart from one structure, the backbone RMSD values for these comparisons are ≤1 Å (Supplementary Table 32). For the antiparallel α-helical barrel proteins, the seeds, models and experimental structures for sc-apCC-6-LLIA and sc-apCC-8 are very similar (Supplementary Table 32). The outlier is sc-apCC-6-SLLA (Supplementary Table 32), in which the experimental structure and model differ at one of the Ser–Ser (***g–g***) helical interfaces (Supplementary Fig. 23e). Such polar contacts are notoriously difficult to model. For the parallel targets, the experimental structures show minor fraying at the C termini of the inner helices compared with the seeds and models, which appears to improve the packing of the external three-helix bundles (Fig. 4b, Supplementary Table 32 and Supplementary Fig. 67). However, the symmetry of the central parallel helices is maintained. The backbone RMSD values for the repeating helix–turn–helix–turn–helix motifs are ≤0.5 Å (Supplementary Fig. 68), which is expected given the low sequence variation in the loops and the hydrophobic cores of these buttressing helices (Fig. 3b and Supplementary Tables 10, 20, 22 and 24). Along with the solution-phase data presented above, this high level of accuracy between the seeds, design models and experimental structures strongly supports the approach of rationally seeding computational design pipelines.

## Discussion

**Fig. 5:**
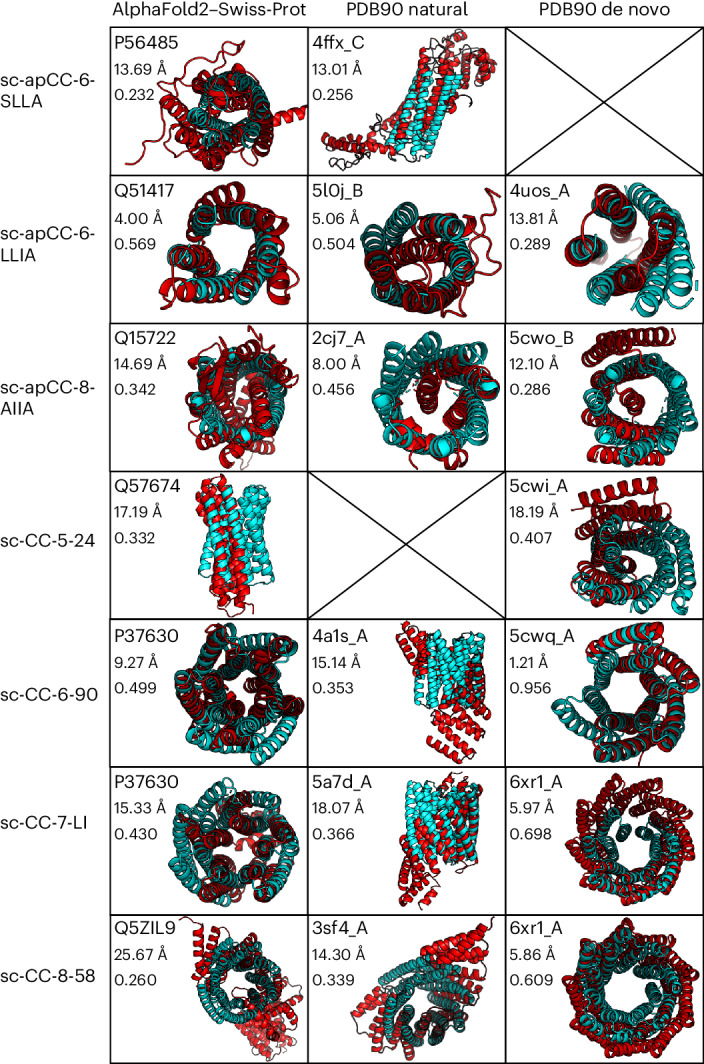
Comparison of de novo α-helical barrel proteins against existing and predicted protein folds. Foldseek^66^ was used for this comparison. Each de novo α-helical barrel protein structure determined in this study (cyan) is overlaid with the top match from the AlphaFold2–Swiss-Prot database,^55,69^ and natural and de novo sequences from the PDB^67,68^ (red). Within each box, the top value is the ID of the matched structure, the middle value is the backbone RMSD between the query and match, and the bottom value is the template modeling score^70^ between the two structures. Source data

In summary, our approach has delivered a set of de novo structures for antiparallel and parallel α-helical barrel proteins with six and eight, and five, six, seven and eight central helices, respectively. We were interested in how similar, if at all, these are to known protein structures and AlphaFold2-predicted models. Therefore, we used them as query structures in Foldseek^66^ to search the RCSB PDB^67,68^ and AlphaFold2–Swiss-Prot databases^55,69^ (Fig. 5, Supplementary Tables 33–46 and Supplementary Fig. 69). This returned natural, de novo and predicted α-helical bundles. However, most of the identified structures and/or models only partially overlapped with our queries, and the sequence identities of the overlapping regions and template modeling scores^70^ were generally low at <20% and ≤0.5, respectively (Supplementary Tables 33–46). Moreover, most have spiraling and/or open structures rather than the cyclically closed structures that we targeted (Fig. 5).

In more detail, for the antiparallel α-helical barrel proteins, sc-apCC-6-SLLA returned partial matches within proteins containing four-helix bundles (Fig. 5 and Supplementary Tables 33 and 34). We found only hypothetical six-helix bundles in the wider UniProt database^55,69^ (for example, UniProt ID, A0A2G8LCW8) (Supplementary Fig. 70). sc-apCC-6-LLIA recovered a four-helix bundle from human vinculin (PDB ID 5L0J)^71^ and a six-helix bundle from the putative transporter protein AmiS from *Pseudomonas aeruginosa* (UniProt ID, Q51417)^55,69^ (Fig. 5 and Supplementary Tables 35 and 36). Socket2 (ref. ^72^) located knobs-into-holes (KIH) interactions indicative of coiled coils in both of these, but only between pairs of helices (Supplementary Fig. 69). sc-apCC-8 yielded mostly poor alignments to helical repeat proteins (Fig. 5 and Supplementary Tables 37 and 38). Interestingly, we found a match to an uncharacterized sequence from *Couchioplanes caeruleus* in UniProt (UniProt ID, A0A3N1FT86) with a putative eight-helix bundle, which again has KIH packing^72^ between pairs of helices (Supplementary Fig. 71).

The parallel designs all showed some similarity with natural and designed helical solenoid proteins (Fig. 5 and Supplementary Tables 39–46). This was anticipated because the helix–turn–helix–turn–helix tertiary fragments used as connectors came from a set of de novo proteins of this type^64^ (Supplementary Table 4). Interestingly, searches with right-handed sc-CC-6, sc-CC-7 and sc-CC-8, but not the left-handed sc-CC-5, consistently returned two hits: the de novo circular tandem repeat protein, cTRP9 (PDB ID, 6XR1)^73^ and the putative inner membrane protein from *E. coli*, YhiM (UniProt ID, P37630)^55,69,74^ (Fig. 5 and Supplementary Tables 39–46). This model, based on five central helices, has the most striking similarity to the parallel α-helical barrel proteins (Fig. 5).

Recently, we expanded the CC+ database of coiled-coil structures to include AlphaFold2 models of 48 proteomes^55,62,69^. Therefore, we searched these for potential single-chain antiparallel and parallel α-helical barrel proteins. This confirmed YhiM and some similar proteins. However, it revealed no further examples of other higher-order antiparallel or parallel-based α-helical barrel proteins in PDB or AlphaFold2 databases. Socket2 (ref. ^72^) analysis of the KIH interactions in the top Foldseek^66^ hits revealed only two- and three-helix coiled-coil bundles, which are unlike the *C*_n_ symmetric coiled-coil barrels with contiguous KIH interactions that we have targeted and made (Supplementary Fig. 69).

Together, these analyses indicate that the de novo α-helical barrel proteins that we present are a new class of single-chain coiled-coil protein. As indicated by dye binding, most of the newly designed proteins have accessible central channels that hit a sweet spot for small-molecule binding and, thus, are ripe for functionalization^20,35–37^. Moreover, the single-chain proteins have a distinct advantage over the oligomeric peptides, as, in principle, the sequence and structural symmetry of the proteins can be broken by mutating residues in individual helices rather than en masse across all helices. Thus, we envisage being able to introduce asymmetric functional sites into the new α-helical barrel proteins. These designs have been achieved through an accessible computational design pipeline that combines rational design principles and readily available computational design and modeling tools. This allowed us to arrive quickly at designed sequences for new coiled-coil-based proteins that surpass the complexity of natural or de novo coiled-coil structures reported to date. Furthermore, this was achieved by testing a small number of gene constructs per target, with high success rates across all designs, which yielded, on average, ~70% soluble peptides and/or proteins with solution-phase biophysical data consistent with the designs (Supplementary Table 47) and resulted in ten (21%) new high-resolution X-ray crystal structures. The solution-phase characterization and high-resolution X-ray structures confirm our targets and, more importantly, our overall strategy of seeding computational design with established and understood rational design rules. We envisage that the accessibility, versatility and robustness of this approach will be of value to others in protein design, leading to applications in synthetic and cell biology, materials science, biotechnology and other areas.

## Methods

### Data analysis

Data were analyzed using Python (v3.8.5), matplotlib (v3.3.2), pandas (v1.1.3) scipy (v1.5.4), seaborn (v0.111.1) and numpy (v1.19.2).

### Computational tools

AlphaFold2 using single-sequence mode and three recycle steps was used to generate models for de novo peptide and protein designs. MASTER^51,52^ was used to build fragments (loops) between adjacent helices in the antiparallel and parallel α-helical barrel assemblies to connect the C termini and N termini of adjacent helices into single polypeptide chains. The Google Colab notebook implementation of loop inpainting using RFDesign^9^ (https://github.com/polizzilab/design_tools) was used to generate short loop sequences (three to seven residues) to span between the different helices of the apCC-Hex backbone. ProteinMPNN^8^ was used to optimize the sequences of the MASTER loops for sc-apCC-8 and parallel protein designs. Additional details of scripts used for computational design from starting scaffold seeds are available in the Zenodo repository (10.5281/zenodo.8277143)^90^ and Woolfson Lab GitHub (https://github.com/woolfson-group/rationally_seeded_computational_protein_design).

### Peptide synthesis

Standard Fmoc automated microwave solid-phase peptide synthesis was performed on a 0.1 mmol scale using a Liberty Blue (CEM) synthesizer with inline ultraviolet (UV) monitoring. Activation was achieved with the coupling reagent *N*,*N’*-diisopropylcarbodiimide (DIC) in *N,N*-dimethylformamide (DMF) (1.0 ml, 1 M) or Oxyma Pure in DMF (1 ml, 0.5 M). Standard deprotections were performed using 20% (v/v) morpholine in DMF at 90 °C for 1 min (125 W for 30 s, 32 W for 60 s). All peptides were manually acetyl capped through the addition of pyridine (0.5 ml) and acetic anhydride (0.25 ml) in DMF (9.25 ml), with shaking at room temperature for 20 min. Peptides were cleaved from the resin with the addition of 10 ml of a mixture of 95:2.5:2.5 (v/v) trifluoroacetic acid (TFA):H_2_O:triisopropylsilane, with shaking at room temperature for 2 h. The TFA solution was then filtered to remove the resin beads and was reduced in volume to ~5 ml or lower using a flow of N_2_. Cleaved peptides were precipitated with cold diethyl ether (~45 ml), isolated using centrifugation and dissolved in a 1:1 mixture of MeCN:H_2_O. Crude peptides were lyophilized to yield a white or off-white powder.

### Peptide purification

All peptides were purified by reverse-phase HPLC (JASCO) using a Luna C18 (Phenomenex) column (150 × 10 mm, 5-μm particle size, 100-Å pore size) on ChromNAV (1.19.01, Build 6). Crude peptides were injected into the column and eluted with a 3 ml min^−1^ linear gradient (40–100%) of MeCN in H_2_O with 0.1% TFA, each over 30 min. Elution of each peptide was detected with inline UV monitoring at 220-nm and 280-nm wavelengths simultaneously. A column oven (50 °C) was used to improve separation. Pure fractions were identified by analytical HPLC and matrix-assisted laser desorption/ionization–time of flight (MALDI–TOF) mass spectrometry. Analytical HPLC traces were obtained using a Jasco 2000 series HPLC system and a Phenomenex Kinetex C18 (100 × 4.6 mm, 5-μm particle size, 100-Å pore size) column. Chromatograms were monitored at 220-nm and 280-nm wavelengths. The linear gradient was 40–100% MeCN in water (each containing 0.1% TFA) over 25 min at a flow rate of 1 ml min^−1^. When required, a column oven (50 °C) was used to assist peptide elution. MALDI–TOF mass spectra were collected on a Bruker UltraFlex MALDI–TOF mass spectrometer operating in positive-ion reflector mode. Peptides were spotted on a ground steel target plate using α-cyano-4-hydroxycinnamic acid dissolved in 1:1 MeCN:H_2_O as the matrix. Masses quoted are for the monoisotopic mass as the singly protonated species.

### Protein expression and purification

All genes were directly cloned into pET28a vectors, transformed and then expressed in *E. coli* Lemo21-DE3 (New England Biolabs). Flasks containing 1 l of Miller’s Luria Broth–kanamycin–chloramphenicol and 0.5 mM l-rhamnose were inoculated with 5 ml of overnight cultures and incubated to an optical density at 600 nm of ~0.6 at 37 °C with 200 r.p.m. shaking. Expression was induced with 0.5 mM isopropyl-β-d-thiogalactoside, and cultures were incubated at 37 °C overnight with 200 r.p.m. shaking. Following expression, cultures were pelleted and resuspended in 20 ml lysis buffer (50 mM Tris, pH 7.4, 500 mM NaCl, 30 mM imidazole, 1 mg ml^−1^ lysozyme) for 30 min at 37 °C. Resuspended pellets were sonicated using a Biologics Model 3000 Ultrasonic homogenizer with settings at 50% power and 90% pulser (1 pulse per second) for 5 min and then clarified at 25,500*g* for 30 min. The clarified lysate was heat shocked at 75 °C for 10 min and then cooled on ice for 10 min before reclarifying at 25,500*g* for 10 min. The expressed proteins were first purified with Ni affinity chromatography at room temperature. Filtered lysate was loaded onto an ÄKTAprime plus (GE, PrimeView 5.31) equipped with a HisTrap HP 5-ml column (Cytiva). His-tagged proteins were eluted using a single step gradient from 0 to 55% buffer B (buffer A consisted of 50 mM Tris, 500 mM NaCl and 30 mM imidazole at pH 7.4; buffer B consisted of 50 mM Tris, 500 mM NaCl and 300 mM imidazole at pH 7.4). Fractions were combined and further purified by SEC using a HiLoad 16/600 Superdex 200-pg size exclusion column (Cytiva) equilibrated in buffer containing 50 mM sodium phosphate and 150 mM NaCl (pH 7.4) at room temperature. Eluted fractions were pooled, concentrated and separated using SDS–PAGE to confirm protein identities.

### Circular dichroism

Circular dichroism data were collected on a JASCO J-810 or J-815 spectropolarimeter fitted with a Peltier temperature controller in the far UV region. Spectra Manager (1.55) was used for data collection. Peptide samples were prepared as 50-μM peptide solutions in PBS (8.2 mM sodium phosphate dibasic, 1.8 mM potassium phosphate monobasic, 137 mM NaCl, 2.4 mM KCl, pH 7.4) at 5 °C. For the antiparallel protein designs, circular dichroism spectra were acquired at a 10-μM protein concentration in PBS at 5 °C. For the parallel protein designs, circular dichroism spectra were acquired at a 5-μM protein concentration at 5 °C. Data were collected in a 1-mm quartz cuvette between wavelengths of 190 nm and 260 nm with the instrument set as follows: band width, 1 nm; data pitch, 1 nm; scanning speed, 100 nm min^−1^; response time, 1 s. Each circular dichroism spectrum was obtained by averaging eight scans and subtracting the background signal of the buffer and cuvette. For thermal response experiments, the circular dichroism signal at a 222-nm wavelength was monitored over the temperature range 5–95 °C at a ramp rate of 60 °C per hour with the same settings and peptide or protein concentrations given above. The spectra were converted from ellipticities (mdeg) to mean residue ellipticities (deg·cm^2^·dmol^−1^·res^−1^) by normalizing for concentration of peptide bonds and the cell path length using the equation

$${\mathrm{MRE}}=\frac{\theta \times {10}^{6}}{c\times l\times n}$$

where the variable *θ* is the measured difference in absorbed circularly polarized light in millidegrees, *c* is the micromolar concentration of the compound, *l* is the path length of the cuvette in millimeters, and *n* is the number of amide bonds in the polypeptide.

### Analytical Ultracentrifugation

AUC was performed on a Beckman Optima X-LA or X-LI analytical ultracentrifuge with an An-50-Ti or An-60-Ti rotor (Beckman-Coulter) equipped with ProteomeLab XL-A (5.5) software. Buffer densities, viscosities, and peptide and protein partial specific volumes (*v̅*) were calculated using SEDNTERP (http://rasmb.org/sednterp). For sedimentation velocity, peptide samples were prepared in PBS at a 150-μM peptide concentration and placed in a sedimentation velocity cell with a two-channel centerpiece and quartz windows. The samples were centrifuged at 50 k.r.p.m. at 20 °C, with a total of 120 absorbance scans taken over a radial range of 5.8–7.3 cm at 5-min intervals. For sedimentation velocity experiments with the antiparallel designs, samples were prepared at a 15-μM protein concentration in PBS. The samples were centrifuged at 50 k.r.p.m. (40 k.r.p.m. for sc-apCC-8) using the same method as the peptide experiments. For sedimentation velocity experiments with the parallel designs, samples were prepared at a 25-μM protein concentration in PBS. The samples were centrifuged at 40 or 50 k.r.p.m. using the same method as the above samples. Data from a single run were fitted to a continuous *c*(*s*) distribution model using SEDFIT (v15.2b)^75^ at a 95% confidence level. Residuals for sedimentation velocity experiments are shown as a bitmap in which the grayscale shade indicates the difference between the fit and raw data (residuals, <−0.05 black and >0.05 white). Good fits are uniformly gray without major dark or light streaks. Sedimentation equilibrium experiments were performed at a 70-μM peptide concentration in 110 μl at 20 °C. The experiment was run in triplicate in a six-channel centerpiece. The samples were centrifuged at speeds in the range 20–45 k.r.p.m., and scans at each recorded speed were duplicated after equilibration for 8 h. Data were fitted using SEDPHAT (v15.2b)^76^ to a single-species model. Monte Carlo analysis was performed to yield 95% confidence limits.

### Ligand binding

Ligand-binding experiments were pipetted in quadruplicate using an epMotion 5070 liquid handler (Eppendorf). The total concentration of ligand was kept constant (1 μM DPH in 5% v/v DMSO), and the concentration of de novo peptide assembly and antiparallel protein design varied from 0 to 30 μM. For parallel designs, ligand concentration was kept constant at 0.5 μM, and the protein concentration was varied from 0 to 24 μM. Data were collected on a Clariostar plate reader (BMG Labtech, 5.40 R3) using an excitation wavelength of 350 nm, and the emission was monitored at 450 nm. Binding constants were extracted by fitting the data to the following equation:

$$y={B}_{\max }\frac{\left(c+x+{K}_{\rm d}\right)+\sqrt{{\left(c+x+{K}_{\rm d}\right)}^{2}-4{cx}}}{2c}$$

where *c* is the total concentration of the constant component (for example, DPH), *x* is the concentration of variable component (for example, peptide or protein), *B*_max_ is the fluorescence signal when all of the constant component is bound and *y* is the fluorescence intensity.

### Size exclusion chromatography small-angle X-ray scattering

Data for single-chain protein designs were obtained at the Diamond Light Source (Didcot, UK) on beamline B21. Samples were prepared to 10 mg ml^−1^ in a 50-mM buffer consisting of sodium phosphate and 150 mM NaCl at pH 7.4. A Superdex 200 Increase 3.2/300 was equilibrated in the same buffer at 4 °C. Buffer subtraction and data merging were performed with Scatter^77^. The first point of the linear Guinier region was *q*_min_, and *q*_max_ was calculated using ShaNum through the ATSAS (3.2.1) interface^78^. MultiFoxS software (Sali Lab, https://github.com/salilab/multifoxs) using a monomer model was used to compare experimental scattering profiles to design models and assess the quality of fit by calculating *χ*^2^ (refs. ^57,58^).

### X-ray crystallography

Diffraction-quality peptide crystals were grown using a sitting-drop, vapor-diffusion method. Commercially available sparse matrix screens were used (Morpheus, JCSG-plus, Structure Screen 1 and 2, Pact Premier and ProPlex from Molecular Dimensions), and the drops were dispensed using a robot (Oryx8, Douglas Instruments). For each well of an MRC 96-well 2-drop plate, 0.3 μl of peptide or protein solution and 0.3 μl of reservoir solution in parallel with 0.4 μl of the peptide or protein solution and 0.2 μl of reservoir solution were mixed, and the plate was incubated at 20 °C. Crystals of antiparallel and parallel protein designs were obtained by optimization using seeding and cross seeding. Crystals were mounted and transferred into a cryogenic solution made of the corresponding reservoir solution supplemented with 25% glycerol and flash cooled in liquid nitrogen.

Diffraction data for the crystals were obtained at the Diamond Light Source on beamlines I04 or I24 (Supplementary Table 30). Data for apCC-Hex-LLIA, apCC-Hex-ALIA collapsed bundle, apCC-Oct-GLIA collapsed bundle, sc-CC-5-24 (MULTIPLEX), sc-CC-6-95 and sc-CC-7-LI were processed using the automated Xia2 pipeline^79^, which ports data through DIALS (2.0.2)^80^ to POINTLESS (1.11.1) and AIMLESS (0.5.32)^81^, as implemented in the CCP4 suite^82^. Data for sc-apCC-6-SLLA, sc-apCC-6-LLIA, sc-apCC-8-AIIA and sc-CC-8-58 were processed through the AUTOPROC pipelines, which use the same integrating and data reduction software in addition to STARANISO^83^. apCC-Hex-LLIA, apCC-Hex-ALIA collapsed bundle, apCC-Oct-GLIA collapsed bundle, sc-apCC-6-LLIA and sc-apCC-8-AIIA were phased using ab initio phasing using ARCIMBOLDO_LITE^44,45^. The initial phases were input into and refined using BUCCANEER^84^. Sc-apCC-6-SLLA, sc-CC-5-24, sc-CC-6-95, sc-CC-7-LI and sc-CC-8-58 were solved by molecular replacement using the AlphaFold2 model for PHASER (2.8.3)^85^. Final structures were obtained after iterative rounds of model building with COOT^86^ and refinement with REFMAC5 (7.1)^87^ and Phenix Refine (1.19.2_4158)^88^. Translation/libration/screw (TLS) parameters were used during refinement as one group per chain for all structures. Torsion noncrystallographic symmetry restraints were used for fragments with a <2 Å RMSD and 90% sequence identity. Solvent-exposed atoms lacking map density were either deleted or left at full occupancy. PISA^82,89^ was used to assess the symmetry of apCC-Hex-LLIA and apCC-Oct-GLIA in which there was one copy of the complete biological assembly in the unit cell, and symmetry operations were required to complete the other copy. This strategy was also used for sc-apCC-6-SLLA in which there was one complete biological assembly in the unit cell, as well as one half of the assembly for which the loops were averaged across the unit cell. The same was also applied for sc-apCC-8-AIIA for two of the eight chains that were found in the unit cell, and a fourfold symmetry operation was used to generate the complete biological assembly. Data collection and refinement statistics are provided in Supplementary Table 30. PISA^82,89^ analyses of all assemblies are provided in Supplementary Table 31.

### Reporting summary

Further information on research design is available in the Nature Portfolio Reporting Summary linked to this article.

## Online content

Any methods, additional references, Nature Portfolio reporting summaries, source data, extended data, supplementary information, acknowledgements, peer review information; details of author contributions and competing interests; and statements of data and code availability are available at 10.1038/s41589-024-01642-0.

### Supplementary information

Supplementary Information Supplementary Methods, Tables 1–47, Figs. 1–71 and references.

Reporting Summary

### Source data

Source Data Fig. 2 PDB and .mtz files used for making figure panels b–d and j–m.

Source Data Fig. 2 Raw data to plot panels e–i.

Source Data Fig. 3 PDB files used for making figure panels h–j.

Source Data Fig. 3 Raw data to plot panels c–g.

Source Data Fig. 4 PDB files and AlphaFold2 models used for making the figure.

Source Data Fig. 5 PDB files and alignments used for figure panels.

Source Data Fig. 5 Raw data used to evaluate Foldseek alignments.

## Data Availability

The PDB, Alphafold2–Swiss-Prot, MASTER^51,52^, CC+ (ref. ^38^) and Foldseek^66^ databases are open source and publicly accessible. ProteinMPNN and AlphaFold2 are open source and publicly accessible. The coordinate and structure factor files for ***g***-***a***-***d***-***e*** = ALIA, ***g***-***a***-***d***-***e*** = GLIA, apCC-Hex, sc-apCC-6-LLIA, sc-apCC-6-SLLA, sc-apCC-8, sc-CC-5-24, sc-CC-6-95, sc-CC-7-LI and sc-CC-8-58 have been deposited in the PDB with accession codes 8QAA, 8QAC, 8QAB, 8QAD, 8QAE, 8QAF, 8QKD, 8QAG, 8QAI and 8QAH, respectively. The raw data and code used in this publication have been deposited in Zenodo (10.5281/zenodo.8277143)^90^ and Woolfson Lab GitHub repositories (https://github.com/woolfson-group/rationally_seeded_computational_protein_design). Source data are provided with this paper.

## Code availability

Code used in this publication for generating figures and for our computational design pipeline is available in the Woolfson Lab GitHub repository (https://github.com/woolfson-group/rationally_seeded_computational_protein_design).
