# Supplementary material for: Rationally seeded computational protein design of ɑ-helical barrels
Source: Nat Chem Biol. 2024 Jun 20;20(8):991–9. doi: 10.1038/s41589-024-01642-0 (PMC11288890; doi:10.1038/s41589-024-01642-0)
Supplement: Supplementary file 2 — Reporting Summary [file 41589_2024_1642_MOESM2_ESM.pdf]

nature portfolio

Corresponding author(s): Derek N Woolfson

Last updated by author(s): Apr 25, 2024

Reporting Summary

Nature Portfolio wishes to improve the reproducibility of the work that we publish. This form provides structure for consistency and transparency in reporting. For further information on Nature Portfolio policies, see our Editorial Policies and the Editorial Policy Checklist.

Statistics

For all statistical analyses, confirm that the following items are present in the figure legend, table legend, main text, or Methods section.

n/a	Confirmed	
☐	☒	The exact sample size (*n*) for each experimental group/condition, given as a discrete number and unit of measurement
☐	☒	A statement on whether measurements were taken from distinct samples or whether the same sample was measured repeatedly
☒	☐	The statistical test(s) used AND whether they are one- or two-sided<br>*Only common tests should be described solely by name; describe more complex techniques in the Methods section.*
☒	☐	A description of all covariates tested
☒	☐	A description of any assumptions or corrections, such as tests of normality and adjustment for multiple comparisons
☐	☒	A full description of the statistical parameters including central tendency (e.g. means) or other basic estimates (e.g. regression coefficient) AND variation (e.g. standard deviation) or associated estimates of uncertainty (e.g. confidence intervals)
☒	☐	For null hypothesis testing, the test statistic (e.g. *F*, *t*, *r*) with confidence intervals, effect sizes, degrees of freedom and *P* value noted<br>*Give P values as exact values whenever suitable.*
☒	☐	For Bayesian analysis, information on the choice of priors and Markov chain Monte Carlo settings
☒	☐	For hierarchical and complex designs, identification of the appropriate level for tests and full reporting of outcomes
☒	☐	Estimates of effect sizes (e.g. Cohen's *d*, Pearson's *r*), indicating how they were calculated

Our web collection on statistics for biologists contains articles on many of the points above.

Software and code

Policy information about availability of computer code

Data collection	Circular dichroism: Jasco 810 or 815, Spectra Manager (1.55). analytical HPLC Jasco 2000 series, ChromNAV (1.19.01 [Build 6]). Analytical ultracentrifugation: ProteomeLab XL-A (5.5). Binding assays: CLARIOstar, Software Version (5.40 R3). FPLC: PrimeView 5.31. Xray crystallography diffraction images were collected on MX-124, MX-104; SEC-SAXS were collected on BL-21 at Diamond Light Source: Diamond light source, UK (https:// www.diamond.ac.uk/).
Data analysis	Analytical ultracentrifugation: SEDFIT (v15.2b), Sedphat (v15.2b). X-ray diffraction data processing and model building: XIA2 (0.5.340-g5578c4a7-dials-1.6) pipeline (utilising AIMLESS (0.5.32), POINTLESS (1.11.1)), XSCALE (Build 20171111), XDS (Build 20200417), Dials (2.0.2), Phenix.phaser (2.8.3), Phenix (1.19.2_4158), CCP4 (7.1), REFMAC (5.8.0267), Coot (0.9.6), ARCHIMBOLDO Lite, PyMOL 2.5.0. PISA, BUCCANEER, AutoPROC, STARANISO (2.4.9), SEC-SAXS: ScAtterIV,ATSAS 3.2.1, MultiFoxs https://github.com/salilab/multifoxs. Data were analysed using Python (3.8.5), matplotlib (3.3.2), pandas (1.1.3), scipy (1.5.4), seaborn (0.11.1), and numpy (1.19.2). All code for data analysis is available in the Zenodo repository (https://doi.org/10.5281/zenodo.8277143) and Woolfson Lab github (https://github.com/woolfson-group/rationally_seeded_computational_protein_design).

For manuscripts utilizing custom algorithms or software that are central to the research but not yet described in published literature, software must be made available to editors and reviewers. We strongly encourage code deposition in a community repository (e.g. GitHub). See the Nature Portfolio guidelines for submitting code & software for further information.

Data

Policy information about availability of data

All manuscripts must include a data availability statement. This statement should provide the following information, where applicable:

- Accession codes, unique identifiers, or web links for publicly available datasets
- A description of any restrictions on data availability
- For clinical datasets or third party data, please ensure that the statement adheres to our policy

Data availability. The ProteinMPNN, AlphaFold2, AlphaFold2-Swissprot, PDB, MASTER, CC+ and Foldseek databases and code are open and publicly accessible. The coordinate files for PDB ids 4ffx, 5l0j, 2cj7, 4a1s, 5a7d, 3sf4, 6xr1, 5cwq, 5cwi, 5cwo, and 4uos were pulled from the PDB after they were found to be matches from the Foldseek database. Alphafold2 models from the af2db-swissprot database for P56485, Q51417, Q15722, Q57674, P37630, and Q5ZIL9 were used after they were found to be matches from the Foldseek database. The coordinate and structure factor files for g-a-d-e = ALIA, g-a- d-e = GLIA, apCC-Hex, sc-apCC-6-LLIA, sc-apCC-6-SLLA, sc-apCC-8, sc-CC-5-24, sc-CC-6-95, sc-CC-7-LI, and sc-CC-8-58 have been deposited in the Protein Data Bank with accession codes 8qaa, 8qac, 8qab, 8qad, 8qae, 8qaf, 8qkd, 8qag, 8qai, and 8qah respectively. The raw data and code used in this publication has been deposited in the Zenodo repository (https://doi.org/10.5281/zenodo.8277143) and Woolfson Lab github (https://github.com/woolfson-group/rationally_seeded_computational_protein_design).

Human research participants

Policy information about studies involving human research participants and Sex and Gender in Research.

Reporting on sex and gender	N/A
Population characteristics	N/A
Recruitment	N/A
Ethics oversight	N/A

Note that full information on the approval of the study protocol must also be provided in the manuscript.

Field-specific reporting

Please select the one below that is the best fit for your research. If you are not sure, read the appropriate sections before making your selection.

☒ Life sciences ☐ Behavioural & social sciences ☐ Ecological, evolutionary & environmental sciences

For a reference copy of the document with all sections, see nature.com/documents/nr-reporting-summary-flat.pdf

Life sciences study design

All studies must disclose on these points even when the disclosure is negative.

Sample size	Biophysical measurements do not have sample sizes but were validated by replication as described below. Moreover, this is not a study where a hypothesis is tested through a statistical analysis of the results/observations of individual datasets. Therefore, issues relevant to statistical hypothesis testing such as sample size do not apply to the experimental data. No sample size calculation was performed, the sample size of 3 was chosen as all replicates contained very similar values because these are highly consistent systems. From three data sets, we were able to calculate the mean and standard deviation of the dataset.
Data exclusions	No data were excluded from this study.
Replication	All attempts at replication of the experiments in this study were successful, and mean and variance values were generated from at least 3 independent measurements in all cases.
Randomization	This study did not involve samples being allocated into experimental groups, and therefore statistical hypothesis issues related to randomisation do not apply to this study.
Blinding	This study does not involve experiments where the outcome would be influenced by blinding, and therefore statistical hypothesis issues related to blinding do not apply to this study.

Reporting for specific materials, systems and methods

We require information from authors about some types of materials, experimental systems and methods used in many studies. Here, indicate whether each material, system or method listed is relevant to your study. If you are not sure if a list item applies to your research, read the appropriate section before selecting a response.

Materials & experimental systems

n/a	Involved in the study
☒	☐ Antibodies
☒	☐ Eukaryotic cell lines
☒	☐ Palaeontology and archaeology
☒	☐ Animals and other organisms
☒	☐ Clinical data
☒	☐ Dual use research of concern

Methods

n/a	Involved in the study
☒	☐ ChIP-seq
☒	☐ Flow cytometry
☒	☐ MRI-based neuroimaging
