# Supplementary figures and images for: Rationally seeded computational protein design of ɑ-helical barrels
Source: Nat Chem Biol. 2024 Jun 20;20(8):991–9. doi: 10.1038/s41589-024-01642-0 (PMC11288890; doi:10.1038/s41589-024-01642-0)

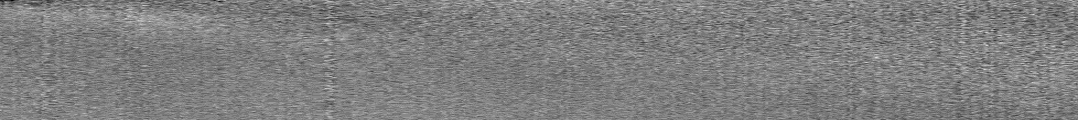

Supplement: Supplementary file 5 — PDB files used for making figure panels h–j. [file 41589_2024_1642_MOESM5_ESM.zip › KIA_RP_RSPD_NatChemBiol_data_Figure3/sc-CC-7-LI_panels_c-g/AUC_panel_e/LI_bit.png]
